# Supplementary material for: Large losses from little lies: Strategic gender misrepresentation and cooperation
Source: PLoS One. 2023 Mar 8;18(3):e0282335. doi: 10.1371/journal.pone.0282335 (PMC9994690; doi:10.1371/journal.pone.0282335)
Supplement: S1 Table — Sample α displays the samples Cronbach’s alpha for multi-item measures. Economics major excludes participants from the Prolific sample as they were not recruited from a student sample, 0 indicates student participants are studying a subject which is not economics, and 1 indicates student participants are studying economics. Steal is a dummy variable where 0 indicates selecting split and 1 indicates selecting steal. Trust is a dummy variable where 0 indicates participants selected “You can’t be too careful” and 1 indicates participants selected “Most people can be trusted”. (DOCX) [file pone.0282335.s001.docx]

**Table S1: Descriptive statistics for study variables**

|  | M | SD | Min | Max | Sample α |
| --- | --- | --- | --- | --- | --- |
| Breakdown of Participants | | | | | |
| U.K. sample | .35 | .48 | 0 | 1 |  |
| Singapore sample | .36 | .48 | 0 | 1 |  |
| Prolific sample | .29 | .45 | 0 | 1 |  |
| Demographic Profiles | | | | | |
| Age | 23.98 | 6.79 | 18 | 68 |  |
| Male | .46 | .50 | 0 | 1 |  |
| Economics major | .07 | .26 | 0 | 1 |  |
| Split vs. Steal and Payout from the Golden Ball game | | | | | |
| Choose to steal | .33 | .47 | 0 | 1 |  |
| Pay out from game | 8.83 | 7.30 | 0 | 20 |  |
| Risk and Trust Behaviours | | | | | |
| Risk attitudes | 5.81 | 2.34 | 0 | 11 |  |
| Trust | .69 | .46 | 0 | 1 |  |
| Dark Traits | | | | | |
| Narcissism | 18.26 | 7.92 | 4 | 36 | .88 |
| Machiavellianism | 14.55 | 7.48 | 4 | 36 | .86 |
| Psychopathy | 11.85 | 6.57 | 4 | 36 | .81 |

Note: Sample α displays the samples Cronbach’s alpha for multi-item measures. Economics major excludes participants from the Prolific sample as they were not recruited from a student sample, 0 indicates student participants are studying a subject which is not economics, and 1 indicates student participants are studying economics. Steal is a dummy variable where 0 indicates selecting split and 1 indicates selecting steal. Trust is a dummy variable where 0 indicates participants selected “You can’t be too careful” and 1 indicates participants selected “Most people can be trusted”.
