# Supplementary material for: Large losses from little lies: Strategic gender misrepresentation and cooperation
Source: PLoS One. 2023 Mar 8;18(3):e0282335. doi: 10.1371/journal.pone.0282335 (PMC9994690; doi:10.1371/journal.pone.0282335)
Supplement: S2 Table — One-way ANOVAs indicate there are no statistical differences in dark triad traits between groups. (DOCX) [file pone.0282335.s002.docx]

**Table S2: Dark Triad Personality by experimental condition**

|  | Control | | True gender | | Randomly assigned opportunity to misrepresent gender | | Randomly assigned gender | |
| --- | --- | --- | --- | --- | --- | --- | --- | --- |
|  | M | SD | M | SD | M | SD | M | SD |
| Narcissism | 18.69 | 7.87 | 19.04 | 7.99 | 17.80 | 8.07 | 18.13 | 7.89 |
| Machiavellianism | 15.52 | 8.28 | 14.48 | 7.09 | 14.28 | 7.51 | 14.60 | 7.56 |
| Psychopathy | 13.04 | 7.25 | 11.58 | 6.03 | 11.72 | 6.83 | 11.78 | 6.67 |

Note: One-way ANOVAs indicate there are no statistical differences in dark triad traits between groups.
