# Supplementary material for: Large losses from little lies: Strategic gender misrepresentation and cooperation
Source: PLoS One. 2023 Mar 8;18(3):e0282335. doi: 10.1371/journal.pone.0282335 (PMC9994690; doi:10.1371/journal.pone.0282335)
Supplement: S3 Table — **<5%. Blind treatment is the reference group. (DOCX) [file pone.0282335.s003.docx]

**Table S3: Marginal effects obtained from Table 3’s multinomial probit model**

| **VARIABLES** | **Both split** | **Split while the other steal** | **Steal while the other split** | **Both steal** |
| --- | --- | --- | --- | --- |
| True gender | 0.008 | -0.001 | 0.044 | -0.051 |
|  | (0.047) | (0.042) | (0.040) | (0.033) |
| Randomised opportunity to misrepresent gender | -0.097** | 0.014 | 0.049 | 0.049 |
|  | (0.045) | (0.038) | (0.030) | (0.030) |
| Randomised gender | -0.048 | -0.033 | 0.033 | 0.047 |
|  | (0.046) | (0.041) | (0.040) | (0.030) |
|  |  |  |  |  |

**Note:** **<5%. Blind treatment is the reference group.
