# Supplementary material for: Large losses from little lies: Strategic gender misrepresentation and cooperation
Source: PLoS One. 2023 Mar 8;18(3):e0282335. doi: 10.1371/journal.pone.0282335 (PMC9994690; doi:10.1371/journal.pone.0282335)
Supplement: S4 Table — **<5%; *<10%. Blind treatment is the reference group. (DOCX) [file pone.0282335.s004.docx]

**Table S4: Marginal effects obtained from Table 4’s multinomial probit model**

| **VARIABLES** | **Both split** | **Split while the other steal** | **Steal while the other split** | **Both steal** |
| --- | --- | --- | --- | --- |
| True gender | 0.007 | 0.046 | 0.044 | -0.051 |
|  | (0.046) | (0.041) | (0.040) | (0.033) |
| **Randomly assigned opportunity to misrepresent gender** |  |  |  |  |
| i) Did not receive opportunity to misrepresent | -0.101** | 0.028 | 0.019 | 0.052 |
|  | (0.050) | (0.044) | (0.043) | (0.033) |
| ii) Randomly assigned opportunity, did not misrepresent | -0.056 | 0.026 | -0.001 | 0.031 |
|  | (0.054) | (0.048) | (0.049) | (0.036) |
| iii) Misrepresented gender | -0.170* | -0.059 | 0.149** | 0.080* |
|  | (0.087) | (0.077) | (0.061) | (0.046) |
| **Randomly assigned gender** |  |  |  |  |
| i) Were not randomly assigned gender | -0.058 | -0.014 | 0.016 | 0.056 |
|  | (0.053) | (0.048) | (0.046) | (0.034) |
| ii) Randomly assigned gender/matched | -0.052 | -0.064 | 0.074 | 0.042 |
|  | (0.064) | (0.059) | (0.053) | (0.040) |
| iii) Randomly assigned gender/mismatched | -0.013 | -0.038 | 0.014 | 0.038 |
|  | (0.068) | (0.055) | (0.060) | (0.043) |
|  |  |  |  |  |

**Note:** **<5%; *<10%. Blind treatment is the reference group.
