# Supplementary material for: Large losses from little lies: Strategic gender misrepresentation and cooperation
Source: PLoS One. 2023 Mar 8;18(3):e0282335. doi: 10.1371/journal.pone.0282335 (PMC9994690; doi:10.1371/journal.pone.0282335)
Supplement: S5 Table — *<10%; ***<1%. Heteroskedasticity-adjusted standard errors at the sessional level are reported in parentheses. The sample consists of those who were randomly allowed the opportunity to misrepresent. Dependent variable is a binary variable: 0 = received an opportunity to misrepresent but did not take it, 1 = misrepresented. The marginal effects are estimated at the means. (DOCX) [file pone.0282335.s005.docx]

**Table S5: Marginal effects from probit regression on the decision to misrepresent**

| **Dependent variable: Choosing to misrepresent gender = 1** |  |
| --- | --- |
| Female matched with male | 0.0177 |
|  | (0.110) |
| Male matched with female | -0.0225 |
|  | (0.0958) |
| Both females | -0.107 |
|  | (0.0921) |
| Age | 0.00721 |
|  | (0.0274) |
| Age-squared | 1.22e-07 |
|  | (0.000349) |
| Take Economics as major (if student) | 0.00489 |
|  | (0.144) |
| Singaporean sample | 0.0719 |
|  | (0.0946) |
| Prolific (U.K. and US) sample | -0.222** |
|  | (0.106) |
| General risk preference | 0.0294* |
|  | (0.0173) |
| Dark triad component: Narcissism | 0.100*** |
|  | (0.0373) |
| Dark triad component: Psychopathy | 0.0243 |
|  | (0.0373) |
| Dark triad component: Machiavellianism | 0.0146 |
|  | (0.0445) |
| Trust | -0.0710 |
|  | (0.0687) |
| Log pseudolikelihood | -83.27 |
| Observations | 166 |

**Note:** *<10%; ***<1%. Heteroskedasticity-adjusted standard errors at the sessional level are reported in parentheses. The sample consists of those who were randomly allowed the opportunity to misrepresent. Dependent variable is a binary variable: 0 = received an opportunity to misrepresent but did not take it, 1 = misrepresented. The marginal effects are estimated at the means.
