# Supplementary material for: Large losses from little lies: Strategic gender misrepresentation and cooperation
Source: PLoS One. 2023 Mar 8;18(3):e0282335. doi: 10.1371/journal.pone.0282335 (PMC9994690; doi:10.1371/journal.pone.0282335)
Supplement: S1 Fig — Text analysis on the 2-minute communication between players across all treatments shows that “split” is the most frequently used words in the conversation. The larger the words are in this word cloud, the more frequent they appeared in the conversation. (DOCX) [file pone.0282335.s006.docx]

**Fig S1: Text analysis of the 2-minute communication across all treatments**

**
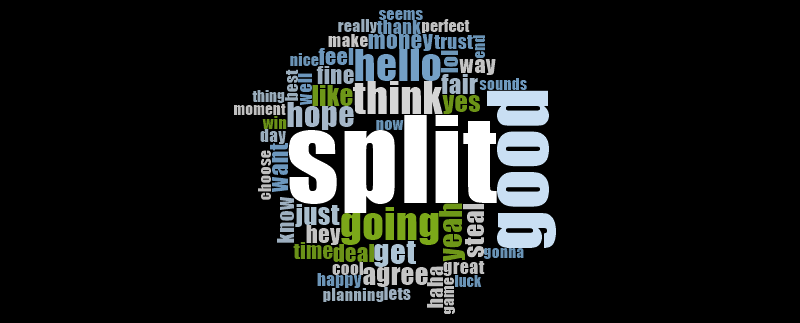
**

**Note:** Text analysis on the 2-minute communication between players across all treatments shows that “split” is the most frequently used words in the conversation. The larger the words are in this word cloud, the more frequent they appeared in the conversation.
