# Supplementary material for: Large losses from little lies: Strategic gender misrepresentation and cooperation
Source: PLoS One. 2023 Mar 8;18(3):e0282335. doi: 10.1371/journal.pone.0282335 (PMC9994690; doi:10.1371/journal.pone.0282335)
Supplement: Attachment — Submitted filename: Referee Report.docx [file pone.0282335.s007.docx]

**Summary**

The paper entitled *Large Losses from Little Lies: Strategic Gender Misrepresentation and Cooperation* investigates an interesting question on cooperation with the possibility of misrepresenting one’s gender. The lab and the field experiment study provide large-sample and multinational evidence that confirm the harmful impact of the available deception on gender. This paper extends mixed previews findings on gender differences in cooperation and consider gender stereotype. Overall, this paper is interesting and helpful in understanding the relationship between small lies on gender and cooperation. However, I have some concerns which may help during the revision.

**Major Comments**

1. What are the purpose and settings of the fourth treatment- Randomly assigned gender? Does this treatment involve deception on subjects? Even if not, I think this design is counterfactual and unreasonable. It is almost impossible to force people to misrepresent their gender in reality. So, not surprisingly, the result of the treatment is disappointing. By contrast, the treatments in regard of hidden gender or gender composition of populations make more sense.
2. There might be some exciting findings in the verbal and anonymous communication before the game. Did the participants have the chance to send gender messages in this part? If so, these messages might be part of the effect of gender misrepresentation or bias in the estimation. Secondly, since this communication is unstructured and participants can talk about whatever they want, does the text data reflects some interesting behavior pattern? For instance, did the participants misrepresenting gender grab this chance to convince their partner to believe them? The authors should consider digging deep into the text data.
3. The consistency between lying behaviors and stealing/splitting behavior should be checked. Some participants might lie to take advantage of stereotypes. However, there might be some participants who were afraid of defection. Once they get the chance to misrepresent, they might lie about their gender to protect themselves from others' defection. This type of participant might be more willing to cooperate. The authors should analyze the different behavior patterns further.
4. One of the most interesting results is the different strategies used by male and female. Which strategy is closer to the rational expectation or which is the more successful strategy? Is there any deeper psychological explanation for this result?
5. Last but not least, the regression coefficients of most treatments are not significant, which means the impacts are small statistically. To make conclusions more convincing, maybe more explanations are needed. What’s more, the analysis on mechanism is somewhat too simple and superficial, it should be more in-depth, for example, including people’s beliefs which are crucial to the results.

**Minor comments**

1. There are several mistakes in the paper. In Introduction section, (p4 line2) "a variant" repeated for 2 times. In Empirical strategy section, p14, paragraph 4, line 3 and 4, letters of the equation failed to be shown, perhaps due to the document export problems.
2. I suggest adding some non-parametric statistical hypothesis tests before skipping into a probit regression, which may help explore differences between two treatments and focus on the significant outcomes. Figure 2 alone is insufficient to clarify the overall treatment effect of four conditions.
3. Wilcoxon Signed Rank in the second paragraph on Page 15 is misused. As mentioned above, the Wilcoxon Rank Sum Test is the test that is used in a between-subject design. It should be checked
4. The reference list are not completed. For example, “Kim (2020)” in the page 14 is not included in the references.
